# New onset of hypomegakaryocytic thrombocytopenia with the potential for progression to aplastic anemia after BNT162b2 mRNA COVID-19 vaccination

**DOI:** 10.1007/s12185-023-03618-7

**Published:** 2023-05-23

**Authors:** Mirei Kobayashi, Akio Mori, Yoshitaka Oda, Emi Yokoyama, Minoru Kanaya, Koh Izumiyama, Makoto Saito, Shinya Tanaka, Masanobu Morioka, Takeshi Kondo

**Affiliations:** 1 Blood Disorders Center, Aiiku Hospital, S4W25, Chuo-ku, Sapporo, 064-0804 Japan; 2 Department of Cancer Pathology, Faculty of Medicine, Hokkaido University, Sapporo, Japan; 3 Institute for Chemical Reaction Design and Discovery (WPI-ICReDD), Hokkaido University, Sapporo, Japan

**Keywords:** Hypomegakaryocytic thrombocytopenia (HMT), Aplastic anemia (AA), COVID-19, SARS-CoV-2, Vaccine

## Abstract

Vaccination with a coronavirus disease-2019 (COVID-19) vaccine is an effective public health measure for reducing the risk of infection and severe complications from COVID-19. However, serious hematological complications after COVID-19 vaccination have been reported. Here, we report a case of new-onset hypomegakaryocytic thrombocytopenia (HMT) with the potential for progression to aplastic anemia (AA) that developed in a 46-year-old man 4 days after the fourth mRNA COVID-19 vaccination. Platelet count rapidly decreased after vaccination and white blood cell count declined subsequently. Bone marrow examination immediately after disease onset showed severely hypocellular marrow (cellularity of almost 0%) in the absence of fibrosis, findings that were consistent with AA. Since the severity of pancytopenia did not meet the diagnostic criteria for AA, the patient was diagnosed with HMT that could progress to AA. Treatment with eltrombopag and cyclosporine was started immediately after diagnosis and cytopenia improved. Although it is difficult to determine whether the post-vaccination cytopenia was vaccine induced or accidental because the association was chronological, vaccination with an mRNA-based COVID-19 vaccine may be associated with development of HMT/AA. Therefore, physicians should be aware of this rare, but serious adverse event and promptly provide appropriate treatment.

**Supplementary Information:**

The online version contains supplementary material available at 10.1007/s12185-023-03618-7.

## Introduction

Aplastic anemia (AA) is a syndrome characterized by a decrease in all blood cells in peripheral blood (pancytopenia) and a decrease in bone marrow cell density (hypoplasia) [1]. Some patients with hypomegakaryocytic thrombocytopenia (HMT) do not meet the diagnostic criteria for AA and that can potentially progress to AA [2].

Severe acute respiratory syndrome coronavirus-2 (SARS-CoV-2) has caused a global pandemic of coronavirus disease-2019 (COVID-19). COVID-19 vaccination is an effective public health measure to reduce the risk of infection and severe complications from COVID-19 [3–5]. Recent studies have shown that some patients developed AA after COVID-19 vaccination. Although the severity of those cases varied, there has been no reported case of HMT, and the mechanisms of the pathogenesis of these diseases remain unclear [6–11]. Here, we present a case of new-onset HMT with the potential for progression to AA after the fourth mRNA COVID-19 vaccination.

## Case report

A 46-year-old Japanese man had been receiving treatment from his family doctor for coronary angina pectoris and dyslipidemia for 7 months. He was prescribed antihypertensive and lipid-lowering medications and his medications had not been changed for more than 6 months. He received his primary (BNT162b2) and third (mRNA-1273) doses of an mRNA-based COVID-19 vaccine at 12 months and 7 months prior to his admission to our hospital, respectively. During this period, he had no cytopenia. He had no history of SARS-CoV-2 infection. He received his fourth dose of vaccine (BNT162b2) 4 days before his admission to our hospital. He experienced chest discomfort in his sleep 2 days after the fourth vaccination and visited a cardiovascular hospital 4 days after the vaccination. Cardiac disease was ruled out, and he was referred to our hospital because thrombocytopenia (platelet count of 2.7 × 10^4^/μL) was observed. He was admitted to our hospital on the same day.

Laboratory data showed a white blood cell count (WBC) of 4200/μL, absolute neutrophil count (ANC) of 2184/μL, red blood cell count (RBC) of 439 × 10^4^/μL, hemoglobin (Hb) of 13.4 g/dL, reticulocyte count of 3.95 × 10^4^/μL, and platelet count of 2.1 × 10^4^/μL (Table 1). Plasma thrombopoietin (TPO) level was 1.04 fmol/ml, which was elevated above the upper limit of the normal range (0.68 fmol/ml). The patient showed no bleeding symptoms at the time of admission. However, his platelet count dropped to 0.9 × 10^4^/μL and he developed a prominent bleeding tendency including nasal bleeding on day 2 of admission.

**Table 1:** Laboratory data on admission

Assay					
WBC	4200/μL	TP	6.7 g/dL	PA-IgG	84 ng/10^7^ cells
Neutro	52%	Alb	3.6 g/dL	ANA	< 40 ×
Lym	34%	Total-Bil	0.6 mg/dL		
Mono	8%	AST	24 U/L	aCL-β2GP1	< 1.2 U/mL
Baso	1%	ALT	30 U/L	Antiphospholipid antibody	4.3 U/mL
Eosino	5%	ALP	68 U/L	High-sensitivity analysis of PNH	
RBC	439 × 10^4^/μL	LDH	174 U/L	Erythrocytes	0.000%
Hb	13.4 g/dL	BUN	17.7 mg/dL	Granulocytes	0.001%
Ht	38.5%	Cr	0.88 mg/dL	C3	116 mg/dL
Plt	2.1 × 10^4^/μL	Haptoglobin	186 mg/dL	C4	22 mg/dL
Reticulocyte	3.951 × 10^4^/μL	CRP	0.29 mg/dL	CH50	39.7 U/mL
Reticulated platelet	7.0%	Ferritin	76.8 ng/mL	TPO	1.04 fmol/mL
PT	11.5 s	Anti-SARS-CoV-2S	19,690 U/mL		
PT–INR	1.00	WT1 mRNA	< 50 copy/μgRNA		
APTT	31.7 s				
Fibrinogen	242 mg/dL				
FDP	< 2.0 μg/mL				

*Anti-SARS-CoV-2S* anti-SARS-CoV-2 spike, *PA-IgG* platelet-associated immunoglobulin G, *ANA* anti-nuclear antibody, *aCL-β2GP1* anticardiolipin β2-glycoprotein-1 complex antibody, *PNH* paroxysmal nocturnal hematuria, *CH* hemolytic complement activity, *TPO* thrombopoietin

**Fig. 1:**
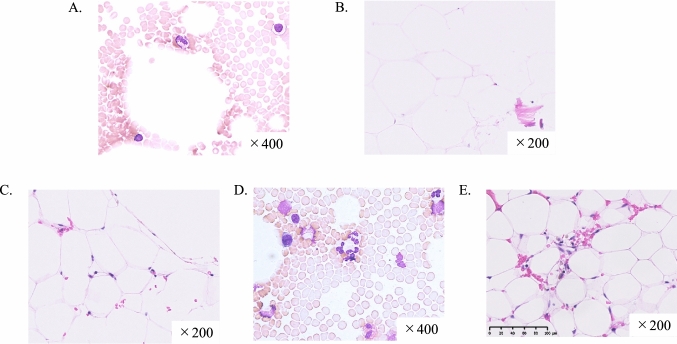
**A** Bone marrow smear for diagnosis of staging by May–Giemsa (M.G.) staging (× 400). It showed hypoplasia and no megakaryocyte proliferation or dysplasia. The number of nucleated cells was 23,000/µL. **B** Bone marrow biopsy for diagnosis of staging by hematoxylin and eosin (H.E.) staging (× 200). It showed severe hypocellular marrow and replacement of hematopoietic cells by fat. Nucleated cell count was 27/mm^2^. **C** Bone marrow biopsy on day 38 at follow-up of staging by H.E. (× 200). It still showed severe hypocellular marrow and marrow cellularity was almost 0%, but the nucleated cell count had increased to 58/mm^2^. **D** Bone marrow smear on day 94 of staging by M.G. (× 400). It still showed hypoplasia, but the number of nucleated cells had increased to 105,000/µL. **E** Bone marrow biopsy on day 94 of staging by H.E. (× 200). It showed slight improvement in cellularity and the nucleated cell count had increased to 84/mm^2^

Since immune thrombocytopenia (ITP) was clinically suspected, high-dose dexamethasone therapy (dexamethasone at a dose of 39.6 mg for 4 days) was immediately started intravenously. He subsequently received transfusions of platelets with a response. Dexamethasone caused a temporary reactive increase in WBC. Bone marrow examination performed on the same day showed severe hypocellular marrow with a cellularity of almost 0% in the absence of fibrosis (Fig. 1A, B). Nucleated cell count was 27 /mm^2^. There was no increase or morphological abnormality of megakaryocytes. There was no increase of blast cells. Bone marrow biopsy of staging by CD61 showed no CD61-positive cells or platelets (Supplemental Fig. 1A). Conventional chromosome analysis showed a normal karyotype. Flow cytometric analysis showed no definite paroxysmal nocturnal hematuria (PNH) clones. These findings were compatible with AA; however, the severity of pancytopenia did not meet the diagnostic criteria for AA [12]. Therefore, he was diagnosed with HMT that could progress to AA and disease severity was considered equivalent to non-severe AA [2, 12].

**Fig. 2:**
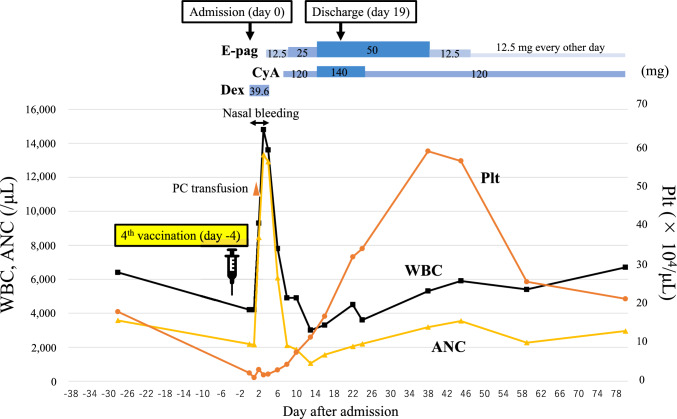
Clinical course in our patient. Platelet count rapidly decreased after vaccination. Dexamethasone caused a temporary reactive increase in white blood cell count, but it declined subsequently. These were gradually restored by administration of eltrombopag and cyclosporine. *WBC* white blood cell count, *ANC* absolute neutrophil count, *Plt* platelet count, *PC* platelet concentrate, *E-pag* eltrombopag, *CyA* cyclosporine, *Dex* dexamethasone

**Fig. 3:**
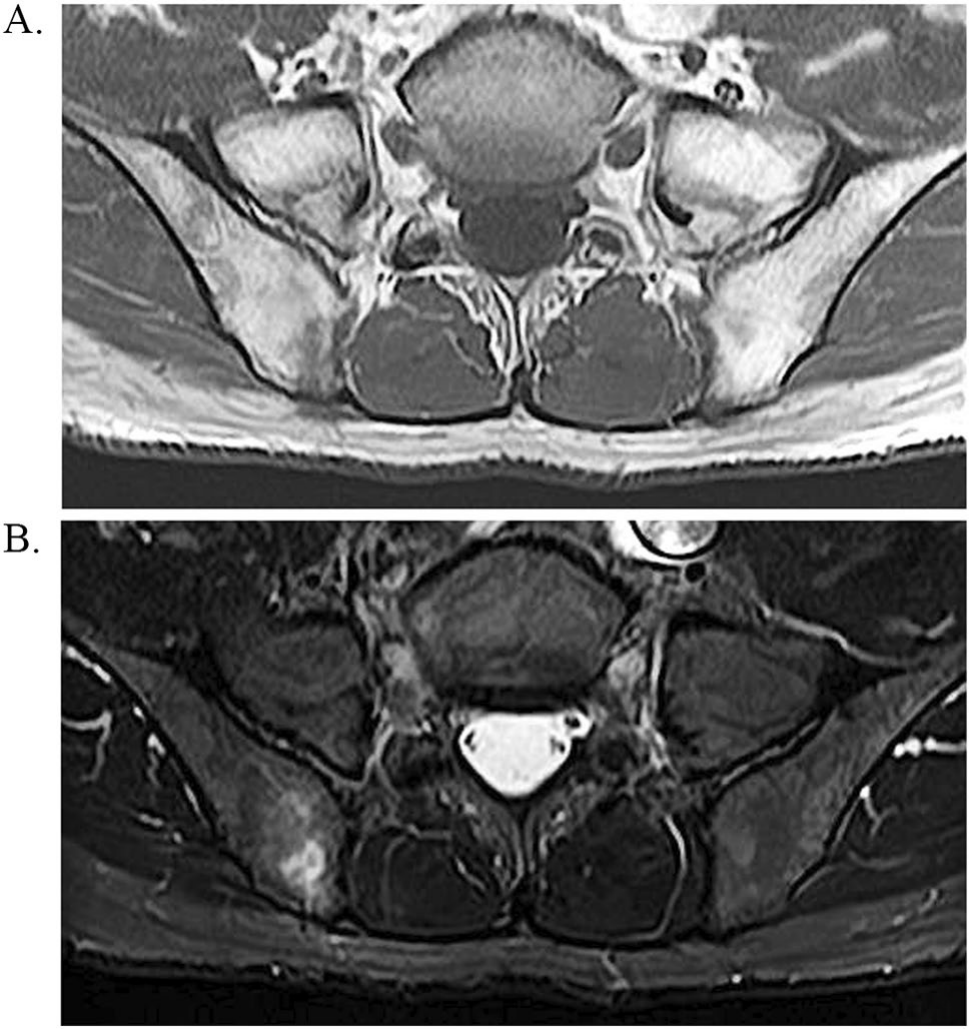
Findings of MRI on day 108. **A** T1-weighted images of the ilium showed diffuse high-intensity areas; however, there were focal low-intensity areas. **B** These low-intensity areas on the T1-weighted images showed high-intensity on the fat-suppressed T2-weighted images

The patient was treated according to the treatment strategies for AA (Fig. 2). The steroid dose was promptly reduced and steroid therapy was discontinued since it was not effective. Instead, eltrombopag at a dose of 12.5 mg/day was administered from day 4 of admission, and the dose was increased to 25 mg/day on day 7. Cyclosporine at a dose of 120 mg/day (2 mg/kg) was also administered from day 6. Platelet count rapidly recovered and was 11.3 × 10^4^/μL on day 13. In contrast, ANC declined gradually and was 1050/μL on day 13 but gradually increased thereafter. He was discharged on day 19. Although his complete blood cell counts were completely recovered, bone marrow biopsy on day 38 still showed severe hypocellular marrow. However, the nucleated cell count had increased to 58/mm^2^ (Fig. 1C). Bone marrow biopsy on day 94 showed a slight improvement in cellularity and the nucleated cell count had increased to 84/mm^2^ (Fig. 1D, E). Furthermore, although there were no CD61-positive cells, CD61-positive platelets were observed on day 38 and 94 (Supplemental Fig. 1B, C). Figure 3 shows the MRI findings on day 108. T1-weighted images of the ilium showed diffuse high-intensity areas; however, there were focal low-intensity areas (Fig. 3A). These low low-intensity areas on the T1-weighted images showed high-intensity on the fat-suppressed T2-weighted images (Fig. 3B). T2-weighted images showed no high-intensity areas suggesting tumor cell infiltration. These findings were consistent with partial recovery of normal hematopoietic cells after treatment.

## Discussion

The development of vaccines against COVID-19 has made it possible to reduce the risk for and severity of SARS-CoV-2 infection; however, concerns about the safety of vaccines continue to be raised [3–5]. Several cases of AA after COVID-19 vaccination including new onset and recurrence with variable severity and various outcomes have been reported [6–11]. Cases of AA that developed after vaccination with either adenoviral-vectored or mRNA vaccine subtypes and after vaccination with the first dose or multiple doses have been reported [6–11]. Historically, vaccines for hepatitis B virus, H1N1 influenza, and varicella-zoster virus have been shown to possibly induce AA [13–17]. Two important mechanisms of depletion of hematopoietic stem cells (HSCs) in AA have been reported: qualitative abnormalities of the HSCs themselves and damage to HSCs by immunologic mechanisms [2, 18]. In patients with AA, immune-mediated destruction of HSCs plays a central role in the pathophysiology, and inflammatory cytokines, such as tumor necrosis factor-α, γ-interferon and transforming growth factor-β, which are secreted from activated immune cells are thought to inhibit hematopoiesis [19–23]. A mechanism that is similar to the mechanism for the development of AA may exist in HMT patients with thrombocytopenia alone, possibly because the levels of myelosuppressive cytokines in the bone marrow of HMT patients are lower than those in AA patients [2].

Some cases of AA after COVID-19 vaccination recovered without treatment, but in most cases, various treatments, including blood transfusion, use of cyclosporine, thrombopoietin receptor agonists, rabbit anti-thymocyte globulin, corticosteroid, and granulocyte colony-stimulating factor and allogeneic hematopoietic stem cell transplantation, were needed [6–11]. It is currently unknown what factors contribute to the differences in prognosis. A recent study showed that patients with HMT that does not meet the diagnostic criteria for AA may potentially progress to AA [2]. Plasma TPO level is useful in assessing megakaryocyte counts, which tend to be higher in AA/HMT and lower in ITP [2, 24]. In this case, plasma TPO level was above the normal range, and considering his bone marrow findings, ITP was not suspected. Furthermore, it was shown that HMT patients with high levels of plasma TPO had better outcomes when treated with cyclosporine [2]. That study revealed that about half of the HMT patients had a pathophysiology similar to that of AA and suggested that early treatment with cyclosporine may improve the prognosis [2]. Measurement of the plasma TPO level might be useful for selecting the appropriate treatment in HMT patients. Although our case showed severe hypocellular marrow that was typical for AA, it did not strictly meet the diagnostic criteria for AA and was diagnosed as HMT. Since the patient’s plasma TPO level at the time of diagnosis was above the normal range, treatment with cyclosporine was considered to have been successful by referring to the previous report [2].

Although it is difficult to determine whether the development of a disease after vaccination was vaccine induced or accidental, various cases of autoimmune disease have been reported after COVID-19 vaccination including autoimmune hepatitis, type 1 diabetes mellitus, acquired hemophilia, ITP, and autoimmune hemolytic anemia as well as aggravation of pre-existing hematologic diseases such as PNH [7, 25–35]. Although the pathogenetic mechanisms by which vaccines cause the development of autoimmune diseases are not yet understood, almost all of the hematologic manifestations after COVID-19 vaccination in previous studies were thought to be related to autoimmune pathways [11]. In addition, there has been no method for predicting the development of autoimmune diseases after vaccination. Previous case reports showed that the initial symptoms related to cytopenia or the first complete blood count abnormalities were merged between the day after vaccination and three months later [6–9]. Before vaccination, our patient had no history of infection, change of medications, or other possible causes of cytopenia. Our patient’s timeline is consistent with that of other previously reported patients. Consequently, this case was most likely to have been COVID-19 vaccine-induced HMT rather than the expression of a pre-existing disease. However, one month has elapsed between the confirmation of a normal platelet count and vaccination. Therefore, we cannot rule out the possibility that thrombocytopenia had already occurred prior to the fourth vaccination. In addition to the accidental onset of the disease, the possibility that the previous vaccination was the cause cannot be ruled out.

It cannot be ruled out that the patient had a transient immunologic mechanism of cytopenia after the fourth vaccination and blood cell counts may have recovered in a few days without treatment. In contrast, anti-TPO receptor antibodies were reported to inhibit the binding of TPO to the TPO receptor and suppress megakaryocyte differentiation in the bone marrow [36]. Anti-TPO receptor antibody-positive cases also existed in ITP patients, with higher TPO levels and poorer responses to TPO receptor agonists [37]. High anti-TPO receptor antibody titers may cause a reactive increase in TPO. In our case, it may be possible that the patient had anti-TPO receptor antibodies, and the platelet count may have recovered rapidly due to the initial steroid therapy, cyclosporine or eltrombopag. Unfortunately, the measurement of anti-TPO receptor antibodies is unavailable in the usual laboratories, therefore, anti-TPO receptor antibodies could not be measured in this case.

In conclusion, vaccination with an mRNA-based COVID-19 vaccine may be associated with the development of HMT/AA and physicians should be aware of this rare but serious adverse event. If symptoms suggestive of cytopenia are merged after COVID-19 vaccination, hematologic evaluation should be performed promptly. Patients with high levels of plasma TPO, as in the present case, are likely to respond to cyclosporine and the use of cyclosporine should be considered as soon as possible. Further studies in large and prospective cohorts are required to elucidate the associations between COVID-19 vaccination and HMT/AA.

## Supplementary Information

Below is the link to the electronic supplementary material.

Supplementary file1 (PDF 127 KB)

## References

[1] Young NS (2018). Aplastic anemia. N Engl J Med.

[2] Saito C, Ishiyama K, Yamazaki H, Zaimoku Y, Nakao S (2016). Hypomegakaryocytic thrombocytopenia (HMT): an immune-mediated bone marrow failure characterized by an increased number of PNH-phenotype cells and high plasma thrombopoietin levels. Br J Haematol.

[3] Polack FP, Thomas SJ, Kitchin N, Absalon J, Gurtman A, Lockhart S (2020). Safety and efficacy of the BNT162b2 mRNA Covid-19 vaccine. N Engl J Med.

[4] Baden LR, Sahly HME, Essink B, Kotloff K, Frey S, Novak R (2021). Efficacy and safety of the mRNA-1273 SARS-CoV-2 vaccine. N Engl J Med.

[5] Dagan N, Barda N, Kepten E, Miron O, Perchik S, Katz MA (2021). BNT162b2 mRNA Covid-19 vaccine in a nationwide mass vaccination setting. N Engl J Med.

[6] Tabata S, Hosoi H, Murata S, Takeda S, Mushino T, Sonoki T (2022). Severe aplastic anemia after COVID-19 mRNA vaccination: causality or coincidence?. J Autoimmun.

[7] Cecchi N, Giannotta JA, Barcellini W, Fattizzo B (2022). A case of severe aplastic anaemia after SARS-CoV-2 vaccination. Br J Haematol.

[8] Röth A, Bertram S, Schroeder T, Haverkamp T, Voigt S, Holtkamp C (2022). Acquired aplastic anemia following SARS-CoV-2 vaccination. Eur J Haematol.

[9] Sridhara S, Nair R, Stanek M (2022). Severe aplastic anemia after receiving SARS-CoV-2 moderna mRNA vaccination. J Hematol.

[10] Chen CY, Chen TT, Hsieh CY, Lien MY, Yeh SP, Chen CC (2023). Case reports of management of aplastic anemia after COVID-19 vaccination: a single institute experience in Taiwan. Int J Hematol.

[11] Woo S, Kim B, Lee SC, Kim MS, Yoon YA, Choi YJ (2022). Very severe immune aplastic anemia after mRNA vaccination against COVID-19 responds well to immunosuppressive therapy: clinical characteristics and comparison to previous reports. Hematology.

[12] Killick SB, Bown N, Cavenagh J, Dokal I, Foukaneli T, Hill A (2016). Guidelines for the diagnosis and management of adult aplastic anaemia. Br J Haematol.

[13] Viallard JF, Boiron JM, Parrens M, Moreau JF, Ranchin V, Reiffers J (2000). Severe pancytopenia triggered by recombinant hepatitis B vaccine. Br J Haematol.

[14] Shenoy KA, Adhikari MRP, Chakrapani M, Shenoy D, Pillai A (2001). Pancytopenia after recombinant hepatitis B vaccine–an Indian case report. Br J Haematol.

[15] Shah C, Lemke S, Singh V, Gentile T (2004). Case reports of aplastic anemia after vaccine administration. Am J Hematol.

[16] Angelini P, Kavadas F, Sharma N, Richardson SE, Tipples G, Roifman C (2009). Aplastic anemia following varicella vaccine. Pediatr Infect Dis J.

[17] Donnini I, Scappini B, Guidi S, Longo G, Bosi A (2012). Acquired severe aplastic anemia after H1N1 influenza virus vaccination successfully treated with allogeneic bone marrow transplantation. Ann Hematol.

[18] Young NS, Calado RT, Scheinberg P (2006). Current concepts in the pathophysiology and treatment of aplastic anemia. Blood.

[19] Nakao S, Takami A, Takamatsu H, Zeng W, Sugimori N, Yamazaki H (1997). Isolation of a T-cell clone showing HLA-DRB1*0405-restricted cytotoxicity for hematopoietic cells in a patient with aplastic anemia. Blood.

[20] Li J, Zhao Q, Xing W, Feng J, Wu H, Li H (2011). Interleukin-27 enhances the production of tumour necrosis factor-alpha and interferon-gamma by bone marrow T lymphocytes in aplastic anaemia. Br J Haematol.

[21] Serio B, Selleri C, Maciejewski JP (2011). Impact of immunogenetic polymorphisms in bone marrow failure syndromes. Mini Rev Med Chem.

[22] Mahgoub IREI, Afify RAA, Botros SKA, Fawzy R (2014). Immunoregulatory cytokines gene polymorphisms in Egyptian patients affected with acquired aplastic anemia. Ann Hematol.

[23] Zeng Y, Katsanis E (2015). The complex pathophysiology of acquired aplastic anaemia. Clin Exp Immunol.

[24] Seiki Y, Sasaki Y, Hosokawa K, Saito C, Sugimori N, Yamazaki H (2013). Increased plasma thrombopoietin levels in patients with myelodysplastic syndrome: a reliable marker for a benign subset of bone marrow failure. Haematologica.

[25] Vuille-Lessard É, Montani M, Bosch J, Semmo N (2021). Autoimmune hepatitis triggered by SARS-CoV-2 vaccination. J Autoimmun.

[26] Patrizio A, Ferrari SM, Antonelli A, Fallahi P (2021). A case of Graves' disease and type 1 diabetes mellitus following SARS-CoV-2 vaccination. J Autoimmun.

[27] Tarawneh O, Tarawneh H (2021). Immune thrombocytopenia in a 22-year-old post Covid-19 vaccine. Am J Hematol.

[28] Radwi M, Farsi S (2021). A case report of acquired hemophilia following COVID-19 vaccine. J Thromb Haemost.

[29] Fattizzo B, Giannotta JA, Cecchi N, Barcellini W (2021). SARS-CoV-2 vaccination in patients with autoimmune cytopenias: The experience of a reference center. Am J Hematol.

[30] Kim G, Choi EJ, Park HS, Lee JH, Lee JH, Lee KH (2021). A case report of immune thrombocytopenia after ChAdOx1 nCoV-19 vaccination. J Korean Med Sci.

[31] Osmanodja B, Schreiber A, Schrezenmeier E, Seelow E (2021). First diagnosis of thrombotic thrombocytopenic purpura after SARS-CoV-2 vaccine-case report. BMC Nephrol.

[32] Gerber GF, Yuan X, Yu J, Cher BAY, Braunstein EM, Chaturvedi S (2021). COVID-19 vaccines induce severe hemolysis in paroxysmal nocturnal hemoglobinuria. Blood.

[33] Murdych TM (2022). A case of severe autoimmune hemolytic anemia after a receipt of a first dose of SARS-CoV-2 vaccine. Int J Lab Hematol.

[34] Portuguese AJ, Sunga C, Kruse-Jarres R, Gernsheimer T, Abkowitz J (2021). Autoimmune-and complement-mediated hematologic condition recrudescence following SARS-CoV-2 vaccination. Blood Adv.

[35] Mori A, Onozawa M, Kobayashi M, Tsukamoto S, Ishio T, Yokoyama E (2022). Humoral response to mRNA-based COVID-19 vaccine in patients with de novo and pre-existing immune thrombocytopenia with exacerbation of thrombocytopenia after vaccination. Br J Haematol.

[36] Kuwana M, Okazaki Y, Kajihara M, Kaburaki J, Miyazaki H, Kawakami Y (2002). Autoantibody to c-Mpl (thrombopoietin receptor) in systemic lupus erythematosus: relationship to thrombocytopenia with megakaryocytic hypoplasia. Arthritis Rheum.

[37] Jing FM, Zhang XL, Meng FL, Liu XM, Shi Y, Qin P (2018). Anti-c-Mpl antibodies in immune thrombocytopenia suppress thrombopoiesis and decrease response to rhTPO. Thromb Res.

