# Supplementary material for: New onset of hypomegakaryocytic thrombocytopenia with the potential for progression to aplastic anemia after BNT162b2 mRNA COVID-19 vaccination
Source: Int J Hematol. 2023 May 23;118(4):477–82. doi: 10.1007/s12185-023-03618-7 (PMC10203663; doi:10.1007/s12185-023-03618-7)
Supplement: Supplementary file 1 — Supplementary file1 (PDF 127 KB) [file 12185_2023_3618_MOESM1_ESM.pdf]

Supplemental Figure 1.

A.

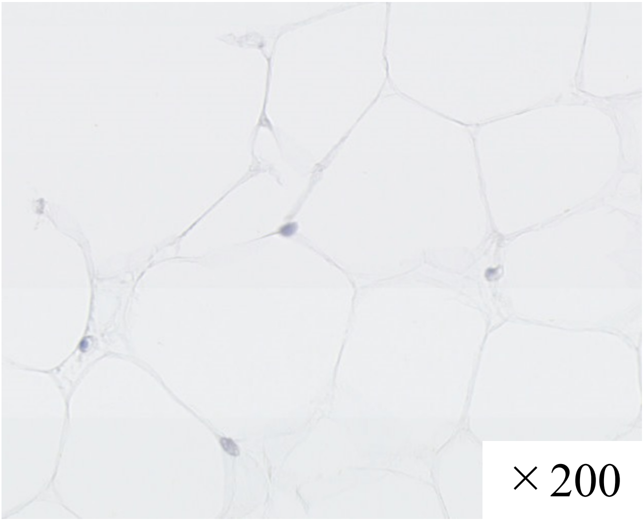

B.

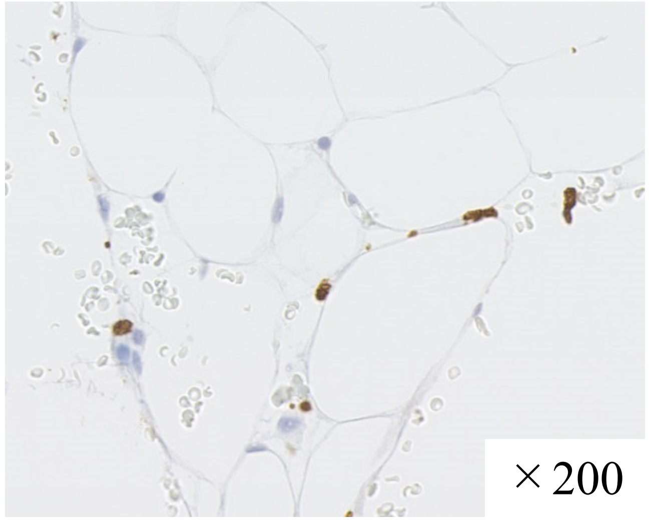

C.

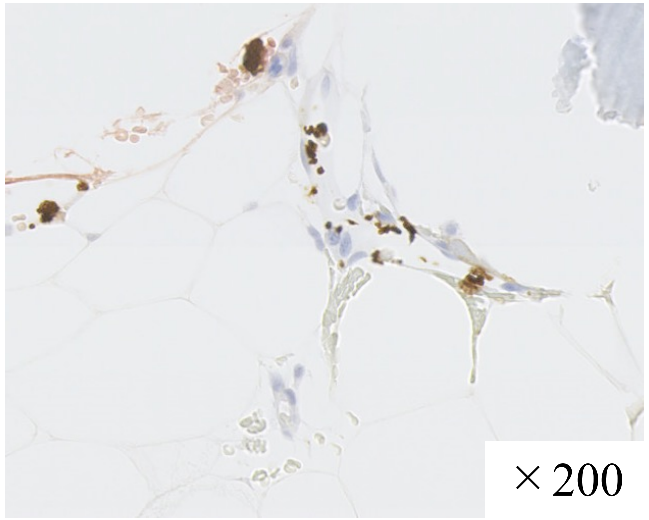

Bone marrow biopsy of staging by CD61 (x200). (A) CD61-positive cells and platelets were not observed at all at the time of initial diagnosis. (B) On day 38, CD61-positive platelets were observed. (C) On day 94, CD61-positive platelets were observed as well as the findings on day 38.
